# A Double‐Edged Sword Perspective on Young Australians' Use of Social Media: A Structured Narrative Review

**DOI:** 10.1002/hpja.70093

**Published:** 2025-09-10

**Authors:** Nasim Salehi, Georgia Rose Marshall, Mohammad Hossein Maziarfar, Alice Zubrinich, Nazanin Madani, Mansoureh Nickbakht, Ahmed A. Moustafa

**Affiliations:** ^1^ Faculty of Health Sciences and Medicine Bond University Gold Coast Australia; ^2^ Iran University of Science & Technology Tehran Iran; ^3^ Southern Cross University Gold Coast Australia; ^4^ Faculty of Humanities University of Cape Town Cape Town South Africa; ^5^ School of Health and Rehabilitation Sciences The University of Queensland Brisbane Australia; ^6^ Faculty of Health Sciences University of Johannesburg Johannesburg South Africa; ^7^ Faculty of Society and Design Bond University Gold Coast Australia

**Keywords:** adolescents, Australia, health promotion, online social networking, social media, wellbeing, young adults

## Abstract

**Issue Addressed:**

Social media's potential use has been underestimated in preventive interventions targeting young people despite its importance in psychosocial development. This structured narrative review examined both the positive and negative use of social media by young Australians and its health impacts with a focus on social media‐based interventions.

**Method:**

Following a narrative review approach, 34 papers were analysed from four databases (Medline, PsycINFO, CINAHL and Embase) from 2010 to 2025 to provide indications for leveraging the positive aspects. Thematic analysis was used for analysing data.

**Results:**

Three key themes emerged as outcomes of young Australians' social media use: (1) the engagement paradox; (2) motivations for social media use (information seeking, identity exploration and social connections); and (3) social media interventions. The positive use of social media by young Australians included using social media for information seeking, social connection and support, as well as finding identity and positive relationships. The negative use of social media included engagement and exposure to harmful content (e.g., substance use), addictive and distractive use of social media, and engaging with body image content. Although a few social media interventions were found, they were identified to be effective through facilitating peer support groups and online discussions on difficult topics.

**Conclusion:**

Understanding the dual impact and use of social media by young Australians will help policymakers and researchers develop interventions that empower young people to effectively navigate social media and maximise its benefits for health and wellbeing. These findings highlight the importance of balancing the benefits and risks of social media. Interventions should focus on fostering safe online spaces and developing resources to address the social and informational needs of young individuals.

## 1 Introduction

### 1.1 Background

Social media platforms have become an essential tool for accessing information, maintaining and exploring connections, and sharing content. Such platforms, like Instagram, TikTok, and Facebook, have largely replaced traditional methods of social connection and have instead introduced the potential for constant connectivity in the form of instant messaging and posting content. Young Australians, defined here as adolescents (11–17 years) and young adults (18–35 years), have emerged within the digital era to include a generation of digital natives. Young Australians spend up to 14+ h a week on social media platforms alone, averaging over 2 h per day [1] and they may experience some form of negative encounter online, such as cyberbullying and inappropriate content exposure [2]. With adolescents and young adults prevailing as the most chronically online demographic with the most adverse health outcomes, the implication of how social media impacts young Australians' health is very significant.

### 1.2 Negative Impacts of Social Media on Young Australians

Since the COVID‐19 pandemic, higher levels of social media engagement have been identified in adolescents and young adults [1]. Subsequently, high levels of social media use have been linked to an increased rate of depression and anxiety [3], loneliness [4], eating disorders [5], and even suicidal ideations [6]. As adolescence and young adulthood are significant developmental periods that lead to major physical and cognitive changes, it has been suggested that engagement on social media during this period can lead to increased vulnerability to psychological deficits [7]. As social media facilitates an environment of unrealistic content and highlight reels, there is growing concern about the impacts of this exposure on adolescents' maturation to young adulthood, their development of identity, and general well‐being [8].

Social media algorithms thrive on user engagement, which can champion extremes and minimise the ordinary and mundane [9]. Erikson [10] emphasised the role of comparison in identity development, a fundamental process during adolescence. As adolescents may seek approval from their desired social groups, they are likely to portray idealised versions of themselves on social media. By exposure and facilitation of unauthentic and/or edited content during adolescence, negative impacts on identity formation into young adulthood and how they engage on social media have been highlighted [11]. For example, exposure to exaggerated content on social media can increase loneliness and social disconnect [4], which is especially detrimental to identity development [12] and in indigenous Australians may reinforce the negative stereotypes [13]. Further, with one in three young people considering themselves socially isolated [4], the implications of the negative impacts of social media cannot be understated. With the total financial loss, alongside psychological impacts, suicide rates in young Australians are estimated to be at $511 million a year [14], highlighting an urgency to prevent the potential negative effects of social media use [15, 16].

### 1.3 Positive Impacts of Social Media on Young Australians

While social media has been subjected to criticism for its negative effects on adolescent and young adults' well‐being, there is evidence that supports its role in self‐discovery, building connections, and health promotion [8]. Although traditional in‐person socialisation is still essential for building significant social bonds [17], social media offers an accessible resource for users to explore connections and autonomy [12, 18]. As adolescence is a formative period for identity and development into young adulthood, social media allows youth who perhaps struggle to form meaningful relationships to find space where they are accepted and supported [18]. Social media platforms include a variety of tools/functions that, if used positively, can enhance the user's experience whilst simultaneously encouraging educational gain, connections, and identity [19]. As young people's main motivation for using social media is generally derived from the desire to form social connections and access information [20], social media presents as a valuable resource for navigating the complexity of adolescence to adulthood, whilst simultaneously having access to support resources.

From a public health perspective, social media has transformed the way in which public health policy providers and government initiatives advocate and advertise health. With social media platforms emerging as individuals' most used source of news and/or information seeking, the advertisement of health initiatives has become more accessible to the general population, including young people. For example, studies suggest that social media interventions are an effective resource in addressing young people's sexual health [21], substance use [22], mental health [23], physical health and exercise [24], among others.

### 1.4 National and International Perspectives

Although several existing international reviews have mapped the positive and negative impacts of social media on adolescent and young adult well‐being [25, 26, 27, 28], there is still a lack of exploration of social media as an intervention for personal growth and well‐being of young populations. For example, such reviews and general peer‐reviewed research have prioritised understanding risk‐based outcomes of social media, such as cyberbullying, depression, and mis/overuse of social media [25, 26, 27], whilst overlooking the potential for health promotion.

Existing reviews on social media use among young people have explored both general youth populations [29] and young Indigenous population [13, 30, 31, 32]. These reviews offer insight into both the negative and positive impacts of social media, including its connection to young people's health, social connectedness, education, and even cultural identity in young Indigenous Australians and sharing positive health messages. However, existing reviews suggest that contemporary social media‐based interventions are often not well designed, evidence based, or widely adopted [29, 30], highlighting a persistent gap between the potential of social media for young people's development, well‐being, and effective implementation in practice. A study in young Indigenous population highlighted co‐creating social media content for effectiveness [13]. Furthermore, the existing reviews previously mentioned are dated before the COVID‐19 pandemic, a fundamental shift in global health that accelerated online engagement and social media use in youth and adults alike.

Aligning with this shift in digital behaviours, Australian government policymakers have actioned a ban for adolescents under the age of 16 from using social media [33]. This ban follows the contemporary belief that social media should be a restricted resource in the hands of youth. However, this approach may not result in long‐term or sustainable outcomes for adolescents' health, education, and subsequently a later generation of young adults. Importantly, a recent study shed some doubts on the impact of social media on youth's mental health in Australia [34]. While it is necessary to address the negatives of social media, it is of greater importance to address its positive applications and subsequently create policy initiatives that empower young Australians' well‐being, social cohesion, belonging, and civic engagement in collaboration with social media. Therefore, identifying the positive implications and interventions of social media in this review, alongside the pitfalls of social media, will provide novel guidance on how to empower young Australians on social media. This provides a more sustainable approach by combining top‐down regulations with a bottom‐up focus on empowerment, ensuring users are equipped to navigate the challenges of the digital world.

### 1.5 Rationale and Objectives

In this structured narrative review, we will focus on the positive and negative aspects of social media and their impact on young Australians (11–35 years). By exploring both aspects of social media use, we aim to provide a holistic scope on how to leverage the positive aspects of social media to empower young Australians development and health whilst informing future public health initiatives. We chose the Australian context because, despite being a developed nation with a world‐renowned healthcare system, there are significant issues related to social media, resulting in various health‐related challenges [35]. Furthermore, considering Australia's ban on social media for adolescents under 16, it is essential to review contemporary research and interventions that highlight how these platforms can be leveraged for positive youth development. Unlike previous reviews that focus primarily on risk identification, this review examines how positive applications can be leveraged within current Australian contexts. Our research therefore aims to:

- Explore the positive and negative impacts of social media among young Australians.
- Understand how social media interventions empower young Australians.

## 2 Methods

### 2.1 Review Aims and Scope

A narrative review was chosen here to allow for broad and flexible exploration of existing literature on social media use and its positive and negative impacts. Further, the synthesis of diverse perspectives and themes will allow the present review to offer a level of novelty required to empower young Australians to inform current health initiatives.

### 2.2 Literature Search Strategy

#### 2.2.1 Identifying the Research Question

To identify relevant research for the review, five popular electronic databases (CIHNAL, Medline, EBSCOhost, ProQuest, and Google Scholar) were used for searching from 2010 to 2025, as social media usage has become more prominent over this decade. The search strategy conducted is as follows: (youth OR adolescent* OR teen* OR “young adult” OR “young person” OR “young people”) AND (“social media” OR “online network*” OR “digital network*” OR “social network*” OR “digital platform”) AND Australia.

### 2.3 Literature Study Selection

Study eligibility criteria included: (a) any social media platforms used (e.g., Facebook, Instagram, Snapchat, TikTok, YouTube, WhatsApp, WeChat, and Telegram), (b) any positive or negative aspects of social media, (c) the positive and negative aspects could be suggested by various perspectives, including youth and young people, parents, and experts, (d) the study's target population must consist of individuals categorised as youth (defined 11–35 in this study), adolescents, teens, young adults, or young people, (e) youth and young individuals may belong to both the general population and at‐risk groups, and the use of social media can encompass various positive strategies; examples include preventive measures, health promotion, mentorship, professional development, job opportunities, peer support, community support, and networking; (f) studies that included multiple countries, including Australia, were considered due to limited information available from the Australian context, and (g) studies must have been published as primary peer‐reviewed papers, conducted in Australia, and reported in the English language. We used Abstract for Medline, CINAHL, PsycINFO and Title or Abstract for Embase for the search. Google Scholar, as well as the references of other included papers, were explored as snowball searching to search for more papers.

### 2.4 Thematic Analysis and Synthesis

**FIGURE 1:**
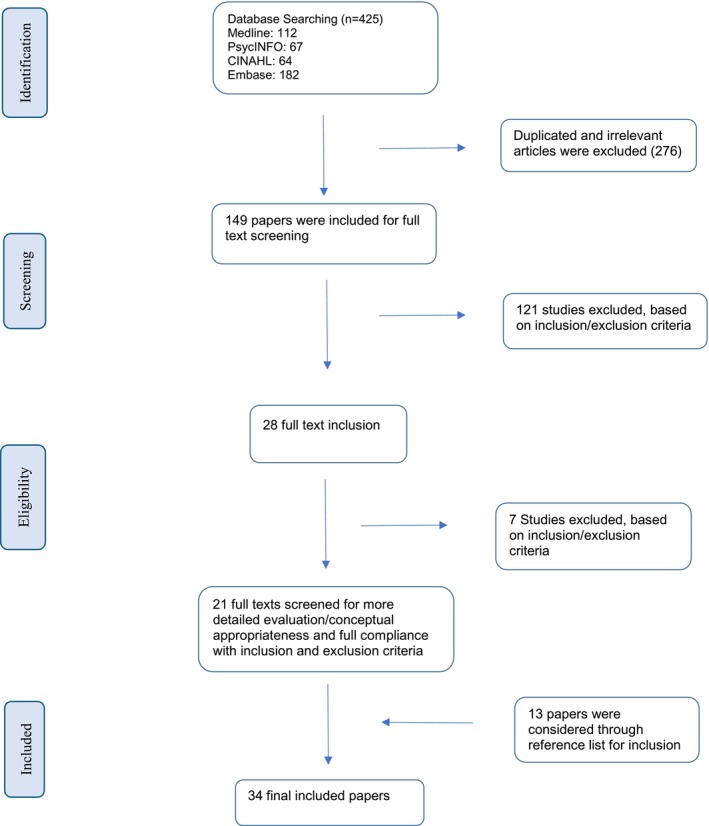
Overview of literature search and selection of the studies.

The lead author NS (an expert in healthcare innovation) screened the initial papers (*n* = 425) to remove duplicates and irrelevant papers. Subsequently, all these papers were divided among the remaining co‐authors (NM and MM) to double‐check inclusion based on predetermined criteria. In cases where uncertainty arose regarding inclusion, a consensus was achieved among the co‐authors. Following the consensus on the final included papers (*n* = 34) (see Figure 1), the five co‐authors (NS, NM, MM, AZ, and GM) conducted data extraction. The data extraction was classified into Authors/year, research objectives, methods, social media platforms used in the studies, outcomes/impact associated with social media use, recommendations, and limitations. MM (an expert in IT and management of technology), MN (an expert in qualitative research in healthcare), and GM (psychology) performed thematic analysis using Braun and Clarke's framework [36, 37]. The researchers acknowledged their backgrounds in technology, psychology, and healthcare research, which may have influenced the interpretation of the findings. Regular team discussions by both researchers and NS helped identify and minimise potential interpretative bias. The first stage of the analysis was to become familiar with the data by reading preliminary extracted data. The second stage was to re‐read the data for generating initial codes by methodically compiling the key information. Thirdly, relevant codes were integrated to generate initial themes. The fourth stage resulted in developing key themes and ensuring that they matched the codes. Then the authors refined and named the themes and prepared the report.

## 3 Findings

An analysis of 34 studies revealed social media use among Australian adolescents and young adults. While the identification of both positive and negative outcomes was present, user experience varied depending on gender, culture, motivations, and contextual factors. Rather than synthesising findings into a dichotomy of positives and negatives, themes were developed inductively to reflect the nuanced ways young Australians use social media. Findings were synthesised into two key themes: (1) the engagement paradox, and (2) motivation for use. To ensure novelty within this narrative review, a third theme was included that addresses (3) social media interventions. In each theme, examples of the positive and negative aspects of social media use were highlighted for adolescents and young adults respectively. A summary of studies included in the review is presented in Table 1.

**TABLE 1:** Summary of publications included in the review of Australian youth using social media.

*N*	References	Research objectives	Sample	Social media platforms used	Key findings
1	[38]	To evaluate the digital platforms used by adolescents for information related to a healthy lifestyle.	297 adolescents; 62.3% female; 13–18 years.	Instagram and Facebook	Using the internet, websites, and social media to find information related to healthy lifestyle and positive behavioural changes (e.g., exercise, weight loss, sleep, and nutrition). However, despite extensive usage, the content may not often be high quality or reliable.
2	[15]	To understand young people's mental health and social media use during the COVID‐19 period. To examine the use of social media for support for suicidal and self‐harm thoughts during COVID‐19 period.	371 young people; 70.4% female; 16–25 years.	Facebook, Instagram, Snapchat, YouTube, TikTok, Twitter, WhatsApp, Reddit, LinkedIn, Tumblr, Pinterest, and Weibo	96% reported using social media once a day; two‐thirds reported an increase in their social media use; and a third used social media for help seeking to support suicidal thoughts/self‐harm, as well as providing support. Young people need to be equipped for mutual support on social media, especially during high‐risk periods.
3	[39]	To explore the use of social media to prevent weight gain in young individuals.	33 young adults; 64% female; 18–25 years.	Social media used as a broad term but specifically mentions Facebook	Facebook was identified as the most popular social media platform for providing social support in a healthy lifestyle intervention for young adults, due to its private group capabilities and integration into their daily routines. They prefer support from strangers with common goals over family/friends for open sharing. Initial in‐person meeting and clear expectations facilitate the social media support groups. Reliable coaches provide guidance and motivation.
4	[40]	To explore mental health and substance use‐related behaviours of young social media users that predicted the consumption of three types of health and fitness‐related social media content: weight loss/fitness pages, detox/cleanse pages, and diet/fitness pages.	1001 young people; 72.23% female; 15–29 years.	Facebook, Instagram, and Twitter	Fitness programs on social media platforms were the most popular among teenage girls. More than a third of participants reported using at least one of the three types of health/fitness social media content.
5	[41]	To evaluate an intervention for young people to communicate online about suicide (#chatsafe).	266 young people with suicide/suicide attempt; 77.4% cisgender female; 16–25 years.	Facebook, Snapchat, and Instagram	It seems safe and acceptable to use social media to provide information related to suicide prevention to young people who were exposed to a suicide or suicide attempt.
6	[42]	To explore LGBTIQ+ young people's use of Tumblr	1304 young LGBTIQ people in a survey and 23 in interviews; 45.6% female, 26.5% male, 19.4% nonbinary, 8.6% self‐described; 16–35 years.	Tumblr	Participants' experience of Tumblr uses for their identity, well‐being, and (dis)connection practices can offer important insights for health, education, and community workers engaging with LGBTIQ+ young individuals.
7	[43]	To explore the safety and acceptability of the #chatsafe campaign and the feasibility of delivering this intervention by social media.	189 young people; 16–25 years.	Facebook, Snapchat, Instagram, Twitter and Tumblr.	Participants found the #chatsafe intervention acceptable and perceived safety when communicating about suicide on social media.
8	[44]	To understand the association between exposure to social media alcohol marketing and alcohol use among young people in Australia and India.	631 participants (330 in India, 301 in Australia), 48.34% female; 13–25 years.	Facebook, YouTube, and Twitter	Engagement with alcohol‐related content on social networking sites was linked to alcohol use behaviours. These behaviours vary by country.
9	[45]	To explore the impact of social media use by depressed adolescents on family functioning within the context of a family‐based treatment intervention.	39 Parents (23 Mothers, 16 Fathers) of adolescents.	Social media used as a broad term but specifically mentions Facebook and Instagram	Parents felt that their adolescents' prolonged exposure to social media exposed their children to more stressors/risks.
10	[46]	To investigate the relationship between alexithymia, narcissism, and social anxiety with social media and internet addiction symptoms.	217 young adults; 77% females, 20.3% males, 2.8% other; 18–35 years.	Social media used as a broad term.	Alexithymia, narcissism, and social anxiety predicted symptoms of internet addiction, whereas only narcissism and social anxiety predicted symptoms of social media addiction. The link between alexithymia and internet addiction symptoms does not apply to excessive use of social media.
11	[47]	To examine young drivers' perceptions of deterrent forces for the behaviour of mobile phone use during driving.	60 young drivers in phase 1 (66.7% female), 503 young drivers in phase 2 (59.6% female); 17–25 years.	Snapchat (majority) and Facebook, Twitter, and Instagram	Traditional deterrence variables (e.g., certainty) were not significant, whereas reconceptualised deterrence variables (e.g., punishment avoidance), along with perceived safety, significantly predicted Snapchat use while driving.
12	[48]	To evaluate the effect of exposure to fitspiration TikTok videos on young women's body dissatisfaction, mood, appearance comparison.	120 female; 17–25 years old.	TikTok	Viewing fitspiration TikTok videos resulted in higher state appearance comparison/state negative mood compared to art TikTok videos. However, it did not affect state body dissatisfaction. State appearance comparison mediated the relationship between TikTok videos on both body dissatisfaction and mood, but trait fit ideal internalisation could not moderate these effects.
13	[16]	To investigate Young people's awareness of the timing and placement of gambling advertising on different social media platforms.	111 young people; 40.5% female; 11–16 years.	Wide range of social media.	More than half of the participants reported seeing gambling advertisements on social media. More than a third recalled gambling ads on YouTube before watching sporting or gaming videos.
14	[49]	To explore how sexualized images typically found on social media might influence adolescent girls' mental health.	24 females; 14–17 years.	A range of platforms including Facebook, Instagram, Snapchat	Participants identified body image and negative appearance comparisons as major concerns when viewing others' images on social media.
15	[50]	To explore the impact of sexualized images on social media on the mental health of adolescent girls	28 participants (7 school support service staff, 11 parents, and 10 youth mental health service providers).	Mostly Instagram.	All participant groups reported that sexualized images on social media contributed to poor mental health among girls.
16	[51]	To explore how young people use and perceive social‐media platforms, for receiving and sharing sexual health information.	22 young people; 16–22 years.	A range of social media (e.g., Facebook, YouTube, and Twitter)	Young people are wary of sharing sexual health information on social media due to stigma, privacy concerns, and the potential for embarrassment and ‘drama’ in their social networks. Humorous and non‐intrusive approaches to sexual health promotion on social media may be more effective in engaging young people.
17	[52]	To identify how LGBTQ adolescents use online platforms to build support networks.	30 LGBTQ Adolescents; 14–17 years.	A range of social media (e.g., YouTube, Facebook and Instagram)	LGBTQ adolescents use social media to connect, support, and train each other. However, they may experience discrimination against and within LGBTQ communities.
18	[53]	To explore positive and negative social media experiences among Muslim young people and parents.	48 participants, including 33 young individuals (76% female; 16–22 years) and 15 parents of young people from Muslim backgrounds.	Instagram, Facebook, SnapChat, YouTube, and TikTok	Social media played a significant role in cultural connections among Muslim young people, strengthening their community connections and religious identity. However, youth and parents are worried about how to connect and yet be protected against online risks, racism, and content that may be against their values. There was also a major ‘digital divide’ as parents view social media as a negative distraction while young people perceive it as an essential part of life.
19	[54]	To investigate young people's comfort in receiving information about sexual health through various channels, including social media.	620 participants; 64% female; 16–29 years.	Facebook and Twitter	The majority of participants reported being comfortable accessing sexual health information from websites (85%), followed by a doctor (81%), school (73%), and mainstream media (67%). Only a few felt comfortable getting information from social media; Facebook (52%), apps (51%), text messages (44%), and Twitter (36%).
20	[55]	To evaluate the impact of social media on young adults' dietary behaviours.	234 young adults; 18–24 years.	Did not limit information gathering to specific social media platforms.	Four themes have been identified: advertising, peer support, access to influencers and web‐based communities, and exposure to content. Access to web‐based health information and social support was contrasted with exposure to fast‐food advertisements.
21	[56]	To understand the services that speech‐language therapists provide to young people with communication disability to facilitate their social media use.	96 qualified speech‐language therapists in Australia with clients aged 12–16 years old.	A range of platforms, including websites and applications (e.g., Facebook, Instagram, YouTube, Snapchat, TikTok, and WhatsApp)	Young people with communication disabilities live and socialise on social media. But speech‐language therapists may not consistently assess/treat young people's use of social media in their professional practice. Most therapists state a lack of confidence and training to help their clients navigate the complexities of online communication. Upskilling healthcare professionals to leverage social media for service provision is recommended.
22	[57]	To explore how adolescents navigate online lifestyle health information.	32 participants from diverse language background; 56% female, 13–18 years.	A range of platforms, including YouTube, Instagram, Facebook and TikTok.	Users can be active searchers versus passive consumers on social media. The most helpful information was visually appealing, well‐organised, and came from credible sources (look good, real, and relatable). Opinions on lifestyle health information found online were mixed—some reported behaviour changes, while others found certain advice difficult to understand and integrate into their lifestyle.
23	[58]	To compare and contrast the experiences of lesbian and gay people with those of bisexual and pansexual people about their social media use, experiences of harassment and exclusion, and mental health.	1304 LGBTQ+ young people; 25% bisexual and 12% pansexual. 46% female, 27% male, 21% non‐binary, 3% gender‐fluid, and 3% other; 16–25 years.	Facebook, Instagram, Snapchat, Tumblr, and Tinder	Bisexual and pansexual participants reported experiencing more frequent harassment and exclusion on social media compared to their lesbian and gay peers, along with poorer mental health outcomes.
24	[59]	To examine the impact of social networking tools/sites on youth's social and emotional well‐being.	1037 young individuals; 49% females; 11–18 years.	Mostly Facebook	Social networking sites offer an important platform for building social connections across various groups.
25	[60]	To explore the opportunities and challenges of using social media and social network sites for sexual health communication with young people in Australia.	22 participants; 50% female; 16–22 years.	Wide range of platforms.	Young people raised concerns about sexual health stigma, privacy, and bullying when using social media for sexual health. Interactivity and peer‐to‐peer sharing on social media were seen as potential benefits for sexual health communication. Young people preferred humorous, engaging content over scary or didactic sexual health messages on social media. Accessibility and media literacy vary among different youth populations, requiring consideration when using social media for sexual health.
26	[61]	To evaluate the types of relationship‐focused content of social media that adolescents encounter, and how they perceive its impact on their beliefs about romantic relationships.	16 adolescents; 50% female; 16–19 years.	Mostly Facebook and Instagram.	Participants reported being able to identify unrealistic/incomplete relationship portrayals. Most participants believed social media relationship portrayals influenced young people's views on relationships.
27	[62]	To investigate the link between different types of social media use (frequency vs. investment) and adolescents' psychological adjustment (social self‐concept, self‐esteem, depressed mood), including gender differences.	1819 adolescents in Western Australia; 55% female; 13–17 years.	Social Networking Sites (e.g., Facebook, Bebo and MySpace)	There is a critical distinction between frequency of use and emotional investment in social media. Higher frequency was linked to a positive social self‐concept, while higher investment was linked to negative outcomes such as lower self‐esteem, higher depressed mood. Simply having a profile was linked to poorer adjustment for females but a higher social self‐concept for males.
28	[63]	To experimentally investigate the impact of Instagram #fitspiration images on young women's mood, body image, and actual exercise behaviour.	108 female university students in, South Australia; 17–25 years.	Instagram	Viewing #fitspiration images increased negative mood and body dissatisfaction, and did not lead to more exercise. Interestingly, it led to a higher perception of workout exertion, indicating a disconnect between psychological impact and physical action.
29	[64]	To qualitatively explore how young people use TikTok for acts of ‘care’ (for self and others), and to identify where the platform's care is lacking.	16 young people in greater Sydney region; 75% female; 13–17 years.	TikTok	TikTok is used by youth for nurturing friendships and self‐care, particularly during pandemic isolation. They experienced a ‘lack of care’ from negative user interactions as well as structural platform issues (e.g., addictive algorithms). They offered practical, constructive solutions to adjust the platform (e.g., enhancing accountability).
30	[32]	To outline a framework and practical considerations for implementing safe and effective online and social media‐based suicide prevention interventions for young people.	Insights from three studies with at‐risk youth in Australia; 14–25 years.	Online platforms and social media (including Facebook)	Online interventions can reduce feelings of isolation by enhancing social connections. Safe implementation is feasible through careful risk protocols and ethical planning. Team‐based human moderation is critical for ensuring user safety and long‐term engagement.
31	[65]	To evaluate the relationship between the duration of leisure internet use and the prevalence of depressive symptoms and psychological distress.	2967 adolescents; 48.4% female; 11–17 years.	The study measured general ‘Internet use for leisure’, which explicitly included social media platforms like Facebook and Twitter.	Very high internet use (≥ 7 h/day) was linked to a doubled risk of depressive symptoms in females and, doubled risk of high psychological distress for both genders.
32	[66]	To explore the lived experience of young adults on social media and its impact on their mental health and well‐being.	An international online survey of 118 young adults, including a large cohort from Australia (26.3%); 18–34 years.	A wide range of social media platforms was discussed, including Facebook, Instagram, YouTube, Twitter, Tumblr, blogs, and gaming platforms.	Social media has a paradoxical impact (amazing as well as horrible). It is an essential tool for connection, support, and finding purpose. However, it can create negative social comparison, anxiety, and unrealistic expectations. The experience is highly personalised and depends on how a person uses each platform.
33	[67]	To understand how young Australians use social media to navigate changing social contexts and experience adulthood.	446 young Australians (70% females) at 24–25 years old (in 2013) and at 29–30 years old (in 2018).	Facebook, Instagram, Twitter, YouTube, WhatsApp, Messenger, and Snapchat	Social media enables connection across distances and busy schedules but increasingly causes negative experiences like time‐wasting, procrastination, and harmful comparisons leading to inferiority and jealousy. Participants suggested more conscious usage by recognizing negative effects and focusing on meaningful activities: information, work, news, and connecting with like‐minded people.
34	[68]	To explore how social media impact the health behaviours of Indigenous Australians.	18 young Indigenous Australians (50% female), aged 17–24 years.	Facebook, Instagram and YouTube	Social media helped connect with family and find inspiration through Indigenous‐specific pages/role models. Online influencers and peers motivated health behaviours, but also created negative impact through increased self‐comparisons

*Note:* All participants of the studies were from Australia except Gupta et al. [44] a collaboration between Australia and India, as well as Dodemaide et al. [66], a collaboration between Australia (with significant participants) and the UK, USA, Germany, Canada, Austria, and South Africa.

### 3.1 The Engagement Paradox

Australian adolescents were found to both actively and passively engage with social media, characterised by scrolling, viewing, liking posts, and interacting in communicative content [57]. Social media can create a paradoxical impact, as the experience is highly personalised, based on the context and depending on how a person uses each platform to create meaning or harm. The platforms can be an essential tool for connection, support, and finding purpose, and at the same time, they can create negative social comparison, anxiety, and unrealistic expectations [66]. Using data from over 400 young Australians, Fu and Cook [67] investigated everyday social media use in this population and found that young Australians engage on social media primarily for social connection. They also found that young Australians have shown an increase in digital literacy around social media use and its negative impacts and changed their focus to a more meaningful usage.

Despite the beneficial engagement, social media can be used addictively [46], such as using Snapchat while driving [47]. Young people who reported high internet use (more than 7 h per day) were twice as likely to be at risk of depression, particularly females, and also had a greater risk of psychological distress in both genders [65]. The influence of social media on adolescents is not just about the frequency of their usage, but how emotionally they invest in it. A study of over 1800 Western Australian youth showed that although frequent use was linked to a stronger social self‐concept, high emotional investment was the main indicator of lower self‐esteem and higher depression rates. This was of particular attention for teenage girls, as simply having a social media account was linked with poorer well‐being, while for boys, it was linked to a more positive social identity [62]. In addition, some young people negatively engaged with social media for fitness inspiration and body image content, which led to unhealthy comparisons when seeing images of others' bodies [49] and fitspiration videos [48]. Fitspiration is a social media trend for inspiring people to improve their health/fitness through diet and exercise, which can be especially detrimental to young women's negative body image perception [48]. The #fitspiration trend may be doing more harm than good, as viewing #fitspiration among young Australian women worsened their mood and body dissatisfaction. Despite the inspirational label, this exposure did not result in more exercise, as they only *perceived* their exertion as higher, although their physical output remained unchanged [63]. Particularly, adolescent girls, when engaging with sexualised and exaggerated body‐image content on social media, found reduced self‐esteem and unrealistic bodily comparisons [50].

### 3.2 Motivations for Social Media Use

Three common factors were identified that influenced social media usage: (i) information seeking, (ii) identity exploration, and (iii) social connection, which were shared across both adolescent and young adult motivations.

- **Information seeking:** Both adolescents and young adults respectively displayed using social media to actively seek health literacy and lifestyle information, such as nutrition, weight management, sleep, and physical activity [38, 39, 40, 57], sexual health [54, 60], and mental health [41]. Among these, Facebook and Instagram were identified as the most popular social media sites for accessing health information and the implementation of lifestyle interventions in both adolescents and young adults alike [39, 40, 43]. Social media use was also found to be a safe and positive resource for young people (16–25 years) to disseminate suicide prevention information [41]. Additionally, positive uses for social media outcomes were displayed in sharing sexual health information using humour, which allowed young people to feel comfortable about obtaining information from social media rather than other resources [42, 54]. Furthermore, accessing healthy lifestyle information was helpful for positive behaviour change [38], with some young people reporting feeling better after seeking support on social media. However, it was also found that others felt worse after providing support to others experiencing suicidal/self‐harm thoughts via social media [15]. In addition, receiving unhealthy information, such as alcohol‐related content on social media, was associated with an increase in young individuals' alcohol use behaviours [44]. In relation to the LGBTQ+ community, young people also found that social media was a positive resource for accessing information and support on sex, safety, and relationships [52]. However, the mismanagement of social media groups had harmful impacts and experiences of discrimination and stigma for LGBTQ young people [52]. Harassment and exclusion were also experienced by bisexual and pansexual participants compared to their lesbian and gay peers [58].
- **Identity exploration:** A study on young Muslims found that social media positively impacted their identity formation, nurtured their sense of belonging, and provided means of cultural connection [53]. Additionally, young adults and adolescents alike used social media to explore sexual orientation (e.g., bisexual and pansexual individuals) and to find a sense of safety in discriminatory situations [42, 52, 58]. For example, social media offered a virtual space for LGBTIQ+ young people to explore their identities and allowed for the formation of a sense of self [52]. Social media sites such as Tumblr were used for connecting LGBTIQ+ young people with others and learning about genders and sexualities, but these connections could be short, anonymous, and sometimes toxic [42]. This exploration was also found to expose adolescents and young adults to the negatives of social media, such as virtual discrimination, harassment, and anti‐LGBTQ conversations [52]. Further, the negative consequences of social media use on self‐perception and identity formation include experiencing body image concerns, increased negative appearance comparisons, and pressure to conform that leads to mental health issues [49], and exposure to unrealistic standards of beauty and success [53]. Specifically for young women, this exposure was especially detrimental to identity formation and development of self‐perception (Papageogiou et al. 2022).
- **Social connections:** Some young people used social media for peer support and building social connections with others [52, 53, 55, 59]. LGBTQ+ adolescents, specifically, used social media to connect, educate, and support each other, although discrimination against and within LGBTQ+ communities still exists in social media [52]. Further, young Australians using social media had positive outcomes in terms of social support and connection, including sharing content and progress, accessing influencers [55], maintaining local and global connections, nurturing friendship, as well as fostering a sense of belonging during COVID‐19 [53, 64]. For example, young Indigenous Australians found social media to positively enhance their ability to socially connect with their community to be inspired by indigenous‐specific pages and role models for adopting a healthy lifestyle; however, it can create destructive social comparison [68]. Although young individuals navigate social media (e.g., TikTok) as a space for self‐care and to nurture friendships, particularly during pandemic isolation, they also face a lack of care from negative user interactions and structural issues such as addictive algorithms [64]. They suggested the platforms be adjusted to give them more control, as well as more accountability from the platform, such as stricter content moderation [64].

### 3.3 Social Media Interventions

Studies that leveraged social media as an effective tool for intervening in young Australians mainly focused on health and well‐being aspects. The #chatsafe campaign [43] targeted young Australians' suicide prevention through randomised control trial. Preliminary evidence found that social media‐based interventions like the #chatsafe campaign facilitated scalable and accessible health promotion using social media's interactive features for online support, such as direct messaging, comment sections, and chats [43]. Health promotion impacts on young Australians' participation in the #chatsafe campaign enhanced self‐efficacy, understanding, and willingness to communicate safely about suicide online [43]. Similarly, [41] social media intervention study further demonstrated that online platforms can effectively target core interpersonal suicide risk factors through fostering social connection and discussion among at‐risk Australian youth. However, they emphasised the need for social media interventions to incorporate systematic safety protocols with continuous human modification for implementation feasibility. Furthermore, a focus group study on young Australians aiming to lose weight found that social media sites, especially Facebook, were effective mediators for enabling social support and motivational peer engagement for change in lifestyle behaviours [39]. Such changes included diet, physical activity, and weight management through scheduled online interactions, posts, discussions, and goal setting via focus group.

## 4 Discussion

This narrative review synthesises 34 studies and identifies a central paradox on social media usage in young Australians—passive versus active engagement, and positive versus negative engagement. This duality is predominant for digital natives who have developed a sense of self from exposure on these platforms. Given this integration, policies focusing solely on simple restriction or banning may not result in a meaningful and sustainable impact. Considering the proposed ban for under‐16s in Australia [69], our review suggests the importance of a nuanced, dual strategy when developing social media policies for young people. One that actively mitigates harm through engaging and interactive training, while simultaneously empowering young people to leverage these powerful platforms for well‐being, belonging, and meaningful civic engagement.

As highlighted by Fardouly [69], there can also be significant enforcement challenges regarding social media banning regulations. Young people can easily navigate around restrictions, shifting towards other platforms such as gaming sites and YouTube. In addition, the banning approach may not provide a quick fix to the fundamental issue of the engagement‐driven algorithms and platform designs that are harmful in the first place. A more sustainable and evidence‐based strategy is required by focusing on comprehensive regulations, including accountability of social media companies to make the platforms safer by design, as well as empowering approaches for parents and children [69].

Our review provides a balanced and timely perspective by examining both the threats and the potential of social media platforms, with a specific focus on the Australian context within current national debates on social media bans. The goal is an actionable reflective roadmap to help government, communities, parents, and schools move beyond simple prohibition and effectively safeguard young people's mental health and digital autonomy. In a world progressively shifting towards technology and AI, the virtual world has become an inevitable part of our lives. Hence, prohibition is not a sustainable strategy. This paper champions a preventative and proactive approach by meeting young people where they are. Rather than designing expensive separate applications, we can potentially integrate trusted support, training content, and empowering approaches onto the social media and digital platforms they use. Our responsibility is not simply to study the harms of social media, but also to actively build digital literacy and resilience among various engaged partners. This can be from parents, school educators, healthcare professionals, and the community to be empowered so that they can also empower young people.

Capturing post‐pandemic data up to 2025, this review moves beyond the simplistic ‘good versus bad’ debate. Instead, it highlights the meaningful and practical pathways that young people engage with social media for positive gain, such as seeking information on mental [41] and sexual health [54, 60], exploring identities [53], and curating social connections [55]. Moreover, our findings highlight social media's vital role in supporting marginalised youth and culturally diverse groups through providing peer support, safety, and promoting a sense of community [13, 52]. Hence, our review indicates that for many at‐risk young people, social media can be an essential support network and an informational support pathway for on‐demand health information. Complementing restriction policies with strategies that enhance digital skills may warrant that while young people are protected, they are also equipped to safely navigate the online world.

A clear pattern emerged among young people, who are using social media as a multifaceted tool. This is of significance to not only young Australians themselves, but also their parents, those working with young people, and governmental bodies. Due to young Australians seeking out health information [38, 39, 40, 57], sexual wellbeing [54, 60], identity formation, finding purpose/meaning, and mental health [41, 66], there exists a unique opportunity for primary health care campaigns to utilise social media intervention to promote evidence‐based information. It was apparent that images or misleading information could lead to poor body image [48, 50], but planned social media interventions such as #chatsafe [43] and strong communities online [39] had profoundly positive impacts. Therefore, planned interventions by government and community bodies could incorporate this content directly into social media platforms that young people already use, making the information more accessible and supportive.

Furthermore, marginalised groups among young Australians were represented in this study as groups who utilised and were impacted by (both positively and negatively) social media use. Social media use allows for building a strong sense of community for these groups, and social media interventions that acknowledge and adopt this information may be beneficial in engaging the more marginalised groups in Australia. For example, young Muslim women found social media an effective tool to build their cultural connections and belonging [53]. Social media interventions that focus not only on disseminating accurate information but also actively aim to foster the sense of community that people within these groups seek, albeit virtual, are more likely to be successful. To produce effective social media interventions for marginalised groups, it needs to be considered that not only does the information need to be integrated with mainstream social media platforms, but it needs to allow active engagement between individuals within these communities. A combination of these factors will likely result in more long‐term changes and maintain the sense of cultural safety these groups often seek.

Overall, our research contributes constructively to the important national conversation on social media and young people. As governments explore regulatory pathways, including potential age‐based restrictions, our work emphasises an opportunity to create a more resilient and sustainable framework. Regulatory measures are most effective when complemented by training and empowerment approaches. Extensive focus solely on prohibition can result in unintended consequences, potentially shifting online activity to less visible and unsupervised environments where young people may have fewer avenues for support and help‐seeking. To achieve the shared goal of developing a safe online environment, we propose a strategic approach that integrates sensible regulation with proactive, preventive, and empowering approaches for confident and responsible digital citizenship. This allows the policy objective to evolve from simple restriction to holistic preparation, ensuring our young people are not only protected but also genuinely empowered and capable.

## 5 Limitations and Future Direction

The current review has some limitations. First, this review is a narrative review rather than a systematic review. Although the process was conducted systematically, it may still be susceptible to selection bias and not provide the totality of existing evidence. Second, the population included a diverse age range from 11 to 35 years, with various developmental phases, and hence different experiences on social media that may require specific interventions. Finally, this review focused exclusively on the Australian young people due to the current policy debate on a social media ban. Therefore, the results may not be generalisable globally.

The studies reviewed also included various limitations that could impact their outcomes and methodological rigour. Many would benefit from enhanced approaches, such as increasing sample sizes to improve the generalisability of findings and employing longitudinal designs to better understand causal relationships between social media use and various positive and negative outcomes. Incorporating control groups would further strengthen result validity, while mixed‐method approaches could provide a more comprehensive view of the phenomena under investigation.

The diversity of study populations also presents a challenge. To capture the varied experiences of social media use among Australian youth, future research should strive for broader demographic representation, including different ethnic groups, socioeconomic status, and geographical locations. Ethical considerations are crucial, necessitating appropriate approvals, privacy protection, and informed consent, especially for younger participants. In addition, addressing limitations related to self‐reported data by integrating objective measures, such as digital tracking tools and biometric assessments, will enhance the reliability and comparability of findings. Addressing these limitations through rigorous research designs will offer a clearer understanding of social media's impact on Australian youth and assist in developing more effective interventions and policies in future research.

Our review suggests several directions for future research. Studies should explore the long‐term effects of social media on various aspects of mental health (including stress, depression, and anxiety), assess the effectiveness of various intervention strategies, and investigate the role of emerging technologies in shaping social media experiences. Adopting interdisciplinary approaches and implementing sector‐wide collaboration will help address the complex challenges of social media use among young Australians.

Considering that the nature of social media access and use is ever‐changing, continued research and funding support are paramount to understand its impact on young Australians, targeting effective interventions and protective practices and policies. Maximising the benefit of social media and mitigating its negative impact using several intervention programs (as discussed above) should be explored extensively in future work. We can promote cyber respect vs. cyberbullying, natural pictures vs. perfect pictures, digital detoxification hour, social responsibility vs. impulsive behaviour, right vs. wrong programs, social respect vs. irresponsible commenting, and so much more. All these interventions are likely to reduce the previously mentioned negative aspects of social media use and leverage the positive aspects.

## 6 Conclusions and Recommendations

This narrative review examined the positive and negative use of social media by young Australians and its health impacts on this population. The positive use included using social media for information seeking, social connection and support, as well as identity and relationships. Negative use of social media includes exposure to harmful content (e.g., gambling and substance use), addictive and distractive use of social media, and engaging with body image content. Our analysis also identified some impacts from using social media for young Australians, including mental health support, social support, informational support, and identity and self‐perception. These findings can help develop effective interventions and policies for young Australians to use social media positively. As identified in the literature review, adolescents are highly sensitive to joining groups and comparing themselves to one another. They are vulnerable to the need for social validation and recognition. Adolescents like to make their own decisions and do not want to be ridiculed or left behind. Therefore, there is a high potential for programs to educate, guide, and empower them.

Although our current review focuses on empowering individuals to navigate social media effectively, it is vital to acknowledge the significant roles of parents/caregivers, schools, healthcare, community, government, and policymakers. At the community level, creating opportunities for positive online interactions and robust support networks can greatly enhance social connections among young people. However, these grassroots efforts must be complemented by top‐down approaches from policymakers and government bodies. Policy‐level actions are crucial for ensuring that social media platforms adopt a safe and supportive environment for young users. For instance, the Australian Online Safety Amendment Act [70], which restricts those under 16 to use social media, demonstrate a clear and well‐intentioned commitment to protecting young people. However, by focusing solely on restriction, we may face challenges with long‐term sustainability and could be enhanced by complementary, empowering strategies. Such restrictions may also impact young Australians' ability to form connections, access information and support, develop their identities, and access new opportunities. It is recommended that governments and policymakers develop interventions that encourage supportive online interactions and engagement on social media sites. School and community programs as well as partnerships with social media platforms can contribute to safer online environments. Additionally, educating parents about the risks and benefits of social media and providing strategies to guide their children's online activities can mitigate negative impacts and encourage positive use. Addressing these broader systemic issues helps create an environment where individual empowerment is supported by strong, effective policies and community initiatives.

Based on our findings, we suggest the following recommendations to raise awareness and promote safe use of social media for different stakeholders.

Government and Policymakers:

1. Health departments should initiate campaigns implemented through partnership with social media influencers and departments of education nationwide. Grants can be provided to create workshops, seminars, theatre plays, posters, modules, as well as to engage influencers to trend the hashtags and promote awareness of both positive and negative sides of social media.
2. Governments should mandate digital mental health training programs within national curriculums for students, teachers and parents about the dark and bright sides of social media. These programs can include workshops to teach how to manage screen time and how to leverage the positive side and not to be impacted by the negative side of it.

Educational Institutions:

3 Educational institutions should implement identity builder workshops, using techniques such as art therapy, role play, and storytelling. Trained educators can use these psychological modalities to help students explore and construct their identity and build up emotional resilience, while they are fostering personal growth in a healthier environment.
4 Educational institutions should develop comprehensive training programs for teachers and parent‐educator sessions focusing on social media risks and benefits, digital literacy, and strategies for supporting online activities.

Non‐Governmental Organisations:

5 Youth‐focused NGOs should create peer‐group support networks nationwide with weekly access, where adolescents can freely discuss their experiences. The leader of these groups should be trained by social workers, counsellors, psychologists, and have regular meetings for feedback.

Technology Developers:

6 Technology companies and developers should create apps with customisable filters that can recognise and block negative and unhealthy content, such as posts related to substance abuse, body image issues, and other harmful material. These apps and filters can motivate creating safer content.
7 Technology developers should create educational apps that adolescents can use to evaluate their social media posts and comments for harmful and unhealthy aspects. These tools should use AI to evaluate the contents based on its tone, harmfulness, and misinterpretation, while promoting constructive online engagement.

Healthcare professionals and researchers:

8 Mental health professionals should integrate social media literacy and digital wellness assessments into routine clinical practice with young people. They should use intervention protocols specific to social media‐related mental health problems.
9 Researchers should conduct studies (e.g., longitudinal studies) to examine the effectiveness of the interventions and their mental health outcomes to provide an evidence base for policy refinement.

## Ethics Statement

The authors have nothing to report.

## Conflicts of Interest

The authors declare no conflicts of interest.

## Data Availability

The data that support the findings of this study are available from the corresponding author upon reasonable request.

## References

[1] Australian Communications and Media Authority (ACMA) , “Communications and Media in Australia: The Digital Lives of Younger Australians,” Australian Government (2021), https://www.acma.gov.au/publications/2021‐05/report/digital‐lives‐younger‐and‐older‐australians.

[2] eSafety Commissioner , “The Digital Lives of Aussie Teens,” (2021), https://www.esafety.gov.au/sites/default/files/2021‐02/The%20digital%20lives%20of%20Aussie%20teens.pdf.

[3] E. B. O'Day and R. G. Heimberg , “Social Media Use, Social Anxiety, and Loneliness: A Systematic Review,” Computers in Human Behavior Reports 3 (2021): 100070, 10.1016/j.chbr.2021.100070.

[4] M. H. Lim , R. Eres , and C. Peck , “The Young Australian Loneliness Survey: Understanding Loneliness In Adolescents and Young Adults,” (2019), https://www.vichealth.vic.gov.au/sites/default/files/The‐young‐Australian‐loneliness‐survey‐Report.pdf.

[5] P. F. Padín , R. González‐Rodríguez , C. Verde‐Diego , and R. Vázquez‐Pérez , “Social Media and Eating Disorder Psychopathology: A Systematic Review,” Cyberpsychology: Journal of Psychosocial Research on Cyberspace 15, no. 3 (2021): 6, 10.5817/CP2021-3-6.

[6] B. Webb , J. C. Looi , S. Allison , N. Bidargaddi , and T. Bastiampillai , “Point of View: Could Social Media Use Be Contributing to Rising Rates of Deliberate Self‐Harm and Suicide in Australian Youth Populations?,” Australasian Psychiatry 30, no. 6 (2022): 694–697, 10.1177/10398562221100093. 35524370

[7] A. Orben , A. Meier , T. Dalgleish , and S. J. Blakemore , “Mechanisms Linking Social Media Use to Adolescent Mental Health Vulnerability,” Nature Reviews Psychology 3 (2024): 407–423, 10.1038/s44159-024-00307-y.

[8] National Academies of Sciences, Engineering, and Medicine (NASEM) , Social Media and Adolescent Health (National Academies Press, 2024), 10.17226/27396. 38713784

[9] J. Whittaker , S. Looney , A. Reed , and F. Votta , “Recommender Systems and the Amplification of Extremist Content,” Internet Policy Review 10, no. 2 (2021): 1–29, 10.14763/2021.2.1565.

[10] E. H. Erikson , Identity and the Life Cycle (WW Norton & company, 1994).

[11] V. Pérez‐Torres , “Social Media: A Digital Social Mirror for Identity Development During Adolescence,” Current Psychology 43, no. 26 (2024): 22170–22180, 10.1007/s12144-024-05980-z.

[12] D. K. Rohit and S. R. M. Prajapati , “Exploring the Impact of Social Media on Identity Formation Among Adolescents,” International Journal of Advanced Research in Science, Communication and Technology (IJARSCT) 3, no. 2 (2023): 516–519, https://ijarsct.co.in/Paper11400E.pdf.

[13] T. Walker , C. Palermo , and K. Klassen , “Considering the Impact of Social Media on Contemporary Improvement of Australian Aboriginal Health: Scoping Review,” JMIR Public Health and Surveillance 5, no. 1 (2019): e11573, 10.2196/11573. 30720442 PMC6379811

[14] I. Kinchin and C. M. Doran , “Correction: The Cost of Youth Suicide in Australia,” International Journal of Environmental Research and Public Health 15 (2019): 672. 10.3390/ijerph15040672 PMC5923714 29617305

[15] E. Bailey , A. Boland , I. Bell , J. Nicholas , L. La Sala , and J. Robinson , “The Mental Health and Social Media Use of Young Australians During the COVID‐19 Pandemic,” International Journal of Environmental Research and Public Health 19, no. 3 (2022): 1077–1093, 10.3390/ijerph19031077. 35162101 PMC8834625

[16] S. L. Thomas , A. Bestman , H. Pitt , et al., “Young People's Awareness of the Timing and Placement of Gambling Advertising on Traditional and Social Media Platforms: A Study of 11–16‐Year‐Olds in Australia,” Harm Reduction Journal 15, no. 1 (2018): 51, 10.1186/s12954-018-0254-6. 30340584 PMC6194705

[17] Z. Wareshallee and S. Choudhary , “Socialising and its Impact on Mental Health,” International Journal of Interdisciplinary Approaches in Psychology 2, no. 10 (2024): 130–155, https://psychopediajournals.com/index.php/ijiap/article/view/567/443.

[18] M. West , S. Rice , and D. Vella‐Brodrick , “Mid‐Adolescents' Social Media Use: Supporting and Suppressing Autonomy,” Journal of Adolescent Research 40, no. 2 (2023): 448–482, 10.1177/07435584231168402.

[19] E. Karahanna , S. X. Xu , Y. Xu , and N. ( A.) Zhang , “The Needs–Affordances–Features Perspective for the Use of Social Media,” MIS Quarterly 42, no. 3 (2018): 737–756, https://www.jstor.org/stable/26635051.

[20] B. Fox , “Loneliness and Social Media: A Qualitative Investigation of Young People's Motivations for Use, and Perceptions of Social Networking Sites,” in Emotions and Loneliness in a Networked Society, ed. B. Fox (Palgrave Macmillan, 2019), 10.1007/978-3-030-24882-6_16.

[21] A. James , J. Power , A. Waling , and G. Lim , “‘We Are Trying to Make Sense of Our Lives’: Health Promotion in the Context of Young People's Digital Sexual Environment,” Health Promotion Journal of Australia 36, no. 2 (2025): e70029, 10.1002/hpja.70029. 40059131 PMC11891117

[22] W. Evans , E. Andrade , S. Goldmeer , M. Smith , J. Snider , and G. Girardo , “The Living the Example Social Media Substance Use Prevention Program: A Pilot Evaluation,” JMIR Mental Health 4, no. 2 (2017): e24, 10.2196/mental.7839. 28655704 PMC5506331

[23] K. P. Kruzan , K. D. A. Williams , J. Meyerhoff , et al., “Social Media‐Based Interventions for Adolescent and Young Adult Mental Health: A Scoping Review,” Internet Interventions 30 (2022): 100578, 10.1016/j.invent.2022.100578. 36204674 PMC9530477

[24] V. A. Goodyear , G. Wood , B. Skinner , and J. L. Thompson , “The Effect of Social Media Interventions on Physical Activity and Dietary Behaviours in Young People and Adults: A Systematic Review,” International Journal of Behavioral Nutrition and Physical Activity 18 (2021): 72, 10.1186/s12966-021-01138-3. 34090469 PMC8180076

[25] P. Best , R. Manktelow , and B. Taylor , “Online Communication, Social Media and Adolescent Wellbeing: A Systematic Narrative Review,” Children and Youth Services Review 41, no. 41 (2014): 27–36, 10.1016/j.childyouth.2014.03.001.

[26] R. Bottaro and P. Faraci , “The Use of Social Networking Sites and Its Impact on Adolescents' Emotional Well‐Being: A Scoping Review,” Current Addiction Reports 9, no. 4 (2022): 518–539, 10.1007/s40429-022-00445-4. 36185594 PMC9516496

[27] E. Bozzola , “The Use of Social Media in Children and Adolescents: Scoping Review on the Potential Risks,” International Journal of Environmental Research and Public Health 19, no. 16 (2022): 1–33, 10.3390/ijerph19169960. PMC9407706 36011593

[28] V. Schønning , G. J. Hjetland , L. E. Aarø , and J. C. Skogen , “Social Media Use and Mental Health and Well‐Being Among Adolescents–A Scoping Review,” Frontiers in Psychology 11 (2020): 1949. 32922333 10.3389/fpsyg.2020.01949 PMC7457037

[29] S. M. Rice , J. Goodall , S. E. Hetrick , et al., “Online and Social Networking Interventions for the Treatment of Depression in Young People: A Systematic Review,” Journal of Medical Internet Research 16, no. 9 (2014): e206, 10.2196/jmir.3304. 25226790 PMC4180352

[30] C. Brusse , K. Gardner , D. McAullay , and M. Dowden , “Social Media and Mobile Apps for Health Promotion in Australian Indigenous Populations: Scoping Review,” Journal of Medical Internet Research 16, no. 12 (2014): e280, 10.2196/jmir.3614. 25498835 PMC4275496

[31] E. S. Rice , E. Haynes , P. Royce , and S. C. Thompson , “Social Media and Digital Technology Use Among Indigenous Young People in Australia: A Literature Review,” International Journal for Equity in Health 15 (2016): 81, 10.1186/s12939-016-0366-0. 27225519 PMC4881203

[32] S. Rice , J. Robinson , S. Bendall , et al., “Online and Social Media Suicide Prevention Interventions for Young People: A Focus on Implementation and Moderation,” Journal of the Canadian Academy of Child and Adolescent Psychiatry 25, no. 2 (2016): 80–86, https://pmc.ncbi.nlm.nih.gov/articles/PMC4879947/. 27274743 PMC4879947

[33] eSafety Commissioner , “Social Media Age Restrictions,” (2024), https://www.esafety.gov.au/about‐us/industry‐regulation/social‐media‐age‐restrictions.

[34] J. A. Blake , A. Sourander , A. Kato , and J. G. Scott , “Will Restricting the Age of Access to Social Media Reduce Mental Illness in Australian Youth?,” Australian and New Zealand Journal of Psychiatry 59, no. 3 (2025): 202–208. 39968662 10.1177/00048674241308692 PMC11837422

[35] J. Fardouly , N. R. Magson , R. M. Rapee , C. J. Johnco , and E. L. Oar , “The Use of Social Media by Australian Preadolescents and Its Links With Mental Health,” Journal of Clinical Psychology 76, no. 7 (2020): 1304–1326. 32003901 10.1002/jclp.22936

[36] V. Braun and V. Clarke , “One Size Fits All? What Counts as Quality Practice in (Reflexive) Thematic Analysis?,” Qualitative Research in Psychology 18, no. 3 (2021): 328–352.

[37] V. Clarke and V. Braun , “Thematic Analysis,” Journal of Positive Psychology 12, no. 3 (2017): 297–298.

[38] M. Armstrong , N. K. Halim , R. Raeside , et al., “How Helpful and What Is the Quality of Digital Sources of Healthy Lifestyle Information Used by Australian Adolescents? A Mixed Methods Study,” International Journal of Environmental Research and Public Health 18, no. 23 (2021): 12844, 10.3390/ijerph182312844. 34886569 PMC8657837

[39] M. Allman‐Farinelli and M. Nour , “Exploring the Role of Social Support and Social Media for Lifestyle Interventions to Prevent Weight Gain With Young Adults: Focus Group Findings,” Journal of Human Nutrition and Dietetics 34 (2021): 178–187, 10.1111/jhn.12774. 32519384

[40] E. R. Carrotte , A. M. Vella , and M. S. Lim , “Predictors of ‘Liking’ Three Types of Health and Fitness‐Related Content on Social Media: A Cross‐Sectional Study,” Journal of Medical Internet Research 17, no. 8 (2015): e205, 10.2196/jmir.4803. 26297689 PMC4642410

[41] L. La Sala , J. Pirkis , C. Cooper , et al., “Acceptability and Potential Impact of the #Chatsafe Suicide Postvention Response Among Young People Who Have Been Exposed to Suicide: Pilot Study,” JMIR Human Factors 10 (2023): e44535, 10.2196/44535. 37204854 PMC10238962

[42] P. Byron , B. Robards , B. Hanckel , S. Vivienne , and B. Churchill , “‘Hey, I'm Having These Experiences’: Tumblr Use and Young People's Queer (Dis)connections,” International Journal of Communication 13 (2019): 2239–2259, https://ijoc.org/index.php/ijoc/article/view/9677.

[43] L. La Sala , Z. Teh , M. Lamblin , et al., “Can a Social Media Intervention Improve Online Communication About Suicide? A Feasibility Study Examining the Acceptability and Potential Impact of the #Chatsafe Campaign,” PLoS One 16, no. 6 (2021): e0253278, 10.1371/journal.pone.0253278. 34129610 PMC8205132

[44] H. Gupta , T. Lam , S. Pettigrew , and R. J. Tait , “The Association Between Exposure to Social Media Alcohol Marketing and Youth Alcohol Use Behaviors in India and Australia,” BMC Public Health 18, no. 1 (2018): 1–11, 10.1186/s12889-018-5645-9. PMC5998576 29895264

[45] A. J. Lewis , T. Knight , G. Germanov , M. L. Benstead , C. I. Joseph , and L. Poole , “The Impact on Family Functioning of Social Media Use by Depressed Adolescents: A Qualitative Analysis of the Family Options Study,” Frontiers in Psychiatry 6 (2015): 131, 10.3389/fpsyt.2015.00131. 26441692 PMC4585291

[46] M. Lyvers , A. Salviani , S. Costan , and F. A. Thorberg , “Alexithymia, Narcissism and Social Anxiety in Relation to Social Media and Internet Addiction Symptoms,” International Journal of Psychology 57, no. 5 (2022): 606–612, 10.1002/ijop.12840. 35262189

[47] V. Truelove , J. Freeman , and J. Davey , “‘I Snapchat and Drive!’ A Mixed Methods Approach Examining Snapchat Use While Driving and Deterrent Perceptions Among Young Adults,” Accident Analysis & Prevention 131 (2019): 146–156, 10.1016/j.aap.2019.06.008. 31255800

[48] S. Pryde and I. Prichard , “TikTok on the Clock but the #Fitspo Don't Stop: The Impact of TikTok Fitspiration Videos on Women's Body Image Concerns,” Body Image 43 (2022): 244–252, 10.1016/j.bodyim.2022.09.004. 36194987

[49] A. Papageorgiou , C. Fisher , and D. Cross , ““Why Don't I Look Like Her?” How Adolescent Girls View Social Media and Its Connection to Body Image,” BMC Women's Health 22, no. 1 (2022): 1–13, 10.1186/s12905-022-01845-4. 35761231 PMC9238066

[50] A. Papageorgiou , D. Cross , and C. Fisher , “Sexualized Images on Social Media and Adolescent Girls' Mental Health: Qualitative Insights from Parents, School Support Service Staff and Youth Mental Health Service Providers,” International Journal of Environmental Research and Public Health 20, no. 1 (2022): 433, 10.3390/ijerph20010433. 36612754 PMC9819033

[51] P. Byron , K. Albury , and C. Evers , “‘It Would Be Weird to Have That on Facebook’: Young People's Use of Social Media and the Risk of Sharing Sexual Health Information,” Reproductive Health Matters 21, no. 41 (2013): 35–44, 10.1016/S0968-8080(13)41686-5. 23684185

[52] M. N. Berger , M. Taba , J. L. Marino , et al., “Corrigendum to: Social Media's Role in Support Networks Among LGBTQ Adolescents: A Qualitative Study,” Sexual Health 18, no. 5 (2021): 421–431, 10.1071/SH21110_CO. 34706814

[53] C. H. Douglass , A. Borthwick , M. S. C. Lim , B. Erbas , S. Eren , and P. Higgs , “Social Media and Online Digital Technology Use Among Muslim Young People and Parents: Qualitative Focus Group Study,” JMIR Pediatrics and Parenting 5, no. 2 (2022): e36858, 10.2196/36858. 35536616 PMC9094717

[54] M. S. Lim , A. Vella , R. Sacks‐Davis , and M. E. Hellard , “Young People's Comfort Receiving Sexual Health Information via Social Media and Other Sources,” International Journal of STD & AIDS 25, no. 14 (2014): 1003–1008, 10.1177/0956462414527264. 24616114

[55] V. J. Friedman , C. J. C. Wright , A. Molenaar , T. McCaffrey , L. Brennan , and M. S. C. Lim , “The Use of Social Media as a Persuasive Platform to Facilitate Nutrition and Health Behavior Change in Young Adults: Web‐Based Conversation Study,” Journal of Medical Internet Research 24, no. 5 (2022): e28063, 10.2196/28063. 35583920 PMC9161050

[56] N. Shelton , N. Munro , M. Keep , J. Starling , and L. Tieu , “Do Speech–Language Therapists Support Young People With Communication Disability to Use Social Media? A Mixed Methods Study of Professional Practices,” International Journal of Language & Communication Disorders 58, no. 3 (2023): 848–863. 36565240 10.1111/1460-6984.12826

[57] R. Raeside , S. S. Jia , J. Redfern , and S. R. Partridge , “Navigating the Online World of Lifestyle Health Information: Qualitative Study With Adolescents,” JMIR Pediatrics and Parenting 5, no. 1 (2022): e35165, 10.2196/35165. 35147506 PMC8881776

[58] R. Nelson , B. Robards , B. Churchill , S. Vivienne , P. Byron , and B. Hanckel , “Social Media Use Among Bisexuals and Pansexuals: Connection, Harassment and Mental Health,” Culture, Health & Sexuality 25, no. 6 (2022): 1–727, 10.1080/13691058.2022.2092213. 35900926

[59] A. Bourgeois , J. Bower , and A. Carroll , “Social Networking and the Social and Emotional Wellbeing of Adolescents in Australia,” Australian Journal of Guidance and Counselling 24, no. 2 (2014): 167–182, 10.1017/jgc.2014.14.

[60] C. Evers , K. Albury , P. Byron , and K. Crawford , “Young People, Social Media, Social Network Sites and Sexual Health Communication in Australia? This Is Funny, You Should Watch It?,” International Journal of Communication 7 (2013): 263–280.

[61] M. Taba , L. Lewis , S. C. Cooper , et al., “What Adolescents Think of Relationship Portrayals on Social Media: A Qualitative Study,” Sexual Health 17, no. 5 (2020): 467–474. 33176904 10.1071/SH20056

[62] C. J. Blomfield Neira and B. L. Barber , “Social Networking Site Use: Linked to Adolescents' Social Self‐Concept, Self‐Esteem, and Depressed Mood,” Australian Journal of Psychology 66, no. 1 (2014): 56–64.

[63] I. Prichard , E. Kavanagh , K. E. Mulgrew , M. S. Lim , and M. Tiggemann , “The Effect of Instagram# Fitspiration Images on Young Women's Mood, Body Image, and Exercise Behaviour,” Body Image 33 (2020): 1–6. 32062021 10.1016/j.bodyim.2020.02.002

[64] J. McLean , C. Southerton , and D. Lupton , “Young People and TikTok Use in Australia: Digital Geographies of Care in Popular Culture,” Social & Cultural Geography 25, no. 5 (2024): 795–813.

[65] E. Hoare , K. Milton , C. Foster , and S. Allender , “Depression, Psychological Distress and Internet Use Among Community‐Based Australian Adolescents: A Cross‐Sectional Study,” BMC Public Health 17, no. 1 (2017): 365. 28449667 10.1186/s12889-017-4272-1 PMC5406913

[66] P. Dodemaide , M. Merolli , N. Hill , and L. Joubert , “Do Social Media Impact Young Adult Mental Health and Well‐Being? A Qualitative Study,” British Journal of Social Work 52, no. 8 (2022): 4664–4683.

[67] J. Fu and J. Cook , “Everyday Social Media Use of Young Australian Adults,” Journal of Youth Studies 24, no. 9 (2021): 1234–1250.

[68] T. Walker , A. Molenaar , and C. Palermo , “A Qualitative Study Exploring What It Means to Be Healthy for Young Indigenous Australians and the Role of Social Media in Influencing Health Behaviour,” Health Promotion Journal of Australia 32, no. 3 (2021): 532–540, 10.1002/hpja.391. 32726490

[69] J. Fardouly , “Potential Effects of the Social Media Age Ban in Australia for Children Younger Than 16 Years,” Lancet Digital Health 7, no. 4 (2025): e235–e236. 40148007 10.1016/j.landig.2025.01.016

[70] N. Fraser and O. Griffiths , “Online Safety Amendment (Social Media Minimum Age) Bill 2024: Bill Digest,” 2024.

